# Cost-Effectiveness Analysis of HLA-B*5801 Testing in Preventing Allopurinol-Induced SJS/TEN in Thai Population

**DOI:** 10.1371/journal.pone.0094294

**Published:** 2014-04-14

**Authors:** Surasak Saokaew, Wichittra Tassaneeyakul, Ratree Maenthaisong, Nathorn Chaiyakunapruk

**Affiliations:** 1 Center of Pharmaceutical Outcomes Research (CPOR), Department of Pharmacy Practice, Faculty of Pharmaceutical Sciences, Naresuan University, Phitsanulok, Thailand; 2 Center of Health Outcomes Research and Therapeutic Safety (Cohorts), School of Pharmaceutical Sciences, University of Phayao, Phayao, Thailand; 3 Pharmacotherapy Outcomes Research Center (PORC), College of Pharmacy, University of Utah, Salt Lake City, Utah, United States of America; 4 Department of Pharmacology, Faculty of Medicine, Khon KaenUniversity, Khon Kaen, Thailand; 5 Research and Diagnostic Center for Emerging Infectious Diseases, Khon Kaen University, Khon Kaen, Thailand; 6 Clinical Pharmacy Research Unit, Department of Clinical Pharmacy, Faculty of Pharmacy, Mahasarakham University, Mahasarakham, Thailand; 7 School of Pharmacy, University of Wisconsin, Madison, Wisconsin, United States of America; 8 School of Pharmacy, Monash University Malaysia, Bandar Sunway, Selangor, Malaysia; 9 School of Population Health, University of Queensland, Brisbane, Australia; Johns Hopkins Bloomberg School of Public Health, United States of America

## Abstract

**Background:**

Stevens-Johnson syndrome (SJS) and Toxic Epidermal Necrolysis (TEN), caused by allopurinol therapy, are strongly associated with the human leukocyte antigen (HLA), HLA-B*5801. Identification of HLA-B*5801 genotype before prescribing allopurinol offers the possibility of avoiding allopurinol-induced SJS/TEN. As there is a paucity of evidence about economic value of such testing, this study aims to determine the cost-effectiveness of HLA-B*5801 testing compared with usual care (no genetic testing) before allopurinol administration in Thailand.

**Methods and Finding:**

A decision analytical and Markov model was used to estimate life time costs and outcomes represented as quality adjusted life years (QALYs) gained. The model was populated with relevant information of the association between gene and allopurinol-induced SJS/TEN, test characteristics, costs, and epidemiologic data for Thailand from a societal perspective. Input data were obtained from the literature and a retrospective database analysis. The results were expressed as incremental cost per QALY gained. A base-case analysis was performed for patients at age 30. A series of sensitivity analyses including scenario, one-way, and probabilistic sensitivity analyses were constructed to explore the robustness of the findings. Based on a hypothetical cohort of 1,000 patients, the incremental total cost was 923,919 THB (USD 29,804) and incremental QALY was 5.89 with an ICER of 156,937.04 THB (USD 5,062) per QALY gained. The cost of gout management, incidence of SJS/TEN, case fatality rate of SJS/TEN, and cost of genetic testing are considered very influential parameters on the cost-effectiveness value of HLA-B*5801 testing.

**Conclusions:**

The genetic testing for HLA-B*5801 before allopurinol administration is considered a highly potential cost-effective intervention in Thailand. The findings are sensitive to a number of factors. In addition to cost-effectiveness findings, consideration of other factors including ethical, legal, and social implications is needed for an informed policy decision making.

## Introduction

Stevens-Johnson syndrome (SJS) and toxic epidermal necrolysis (TEN) are acute life-threatening conditions. SJS is characterized by high fever, malaise and a rapidly developing blistering exanthema of macules and target-like lesion, accompanied by mucosal involvement. TEN has similar presentations with an event more extensive skin detachment and a higher mortality rate [1]. Although the incidence of SJS/TEN is as low as 0.4 to 6 persons per million populations each year [1]–[3], the mortality rate for this condition has been estimated at 5–50% and 30–70% of patients surviving SJS/TEN suffer from long-term sequelae of the disease such as severe dry eye syndrome [4]–[7]. There are many causes of SJS/TEN such as chemical exposures, mycoplasma pneumonia, viral infections, and immunizations but the most common causes are medications (∼80%) [7]. Allopurinol is one of the most frequent drugs associated with SJS and TEN [8], [9].

Allopurinol, an inhibitor of xanthine oxidase, is the most commonly used urate-lowering agent in clinical practice. It is the treatment of choice for urate overproduction or tophaceous gout, nephrolithiasis, and urate nephropathy [10]–[15]. Allopurinol should be started at low dose (100 mg/day) and increased slowly by 100 mg every 2–4 weeks to achieve a serum uric acid level of 6 mg/dl or less (max dose is 800 mg/day). The usual prescribed dose was 300 mg/day [11], [12], [16]. In patients with renal insufficiency, allopurinol was usually started on 50–100 mg/day and gradually titrated until achieve goal of uric acid level [11].

Besides allopurinol, several drugs were used to reduce uric acid levels in gouty patients with hyperuricemia. Febuxostat is a new xanthine oxidase inhibitor with a different chemical class from allopurinol. It is recommended as an alternative for allopurinol-intolerant patients and can be prescribed at unchanged doses for patients with mild-moderate renal or hepatic impairment. However, this drug is available in the US and Europe, but not in some other countries such as Thailand [11], [12], [14], [15]. Probenecid, a uricosuric agent, can be used as an alternative to a xanthine oxidase inhibitor in patients with normal renal function [10]–[12], [14]–[16]. Previous studies reported that probenecid was equivalent to or more effective than allopurinol in gouty patients with normal renal function who excrete uric acid less than 800 mg/24 hours [11], [17]. However, its use is limited by its contraindication in patients with nephrolithiasis and patients with renal insufficiency [10]–[12], [14], [15], [17]. Benzbromarone is another drug for gout management. It is more potent than probenecid and can be used in patients with a creatinine clearance as low as 25 ml/min. However, hepatotoxicity has led to its removal from some markets worldwide but it is still available in Thailand [10], [12].

Although allopurinol is generally well tolerated by most patients, cutaneous adverse reactions to this drug occur in 2% of patients [18]. The cutaneous adverse reactions induced by allopurinol range from mild skin rash to severe cutaneous adverse reactions (SCARs) including Stevens-Johnson syndrome (SJS),toxic epidermal necrolysis (TEN), drug hypersensitivity syndrome or drug reaction with eosinophilia and systemic symptoms (DRESS) [1], [8], [19]. Recently, various studies reported the strong associations of between the genetic marker, HLA-B*5801, and allopurinol-induced SJS/TEN [1], [8], [20]. However, there is a paucity of evidence about the cost-effectiveness of HLA-B*5801 genetic testing before allopurinol administration. The identification of genetic factors predisposing to development of allopurinol-induced SJS/TEN offers the possibility of avoiding this drug in individuals with such susceptibility. Given the serious life-threatening consequences, long-term sequelae after developing SJS/TEN, and the availability of the alternative drugs; pharmacogenetic testing for HLA-B*5801 before allopurinol administration may be justifiable and valuable in preventing SJS/TEN caused by allopurinol. From the policy maker's perspective, information on value for money for this intervention would facilitate informed decision making. This study aims to determine cost-effectiveness of HLA-B*5801 testing before allopurinol administration using societal perspective.

## Methods

### Overview

**Figure 1:**
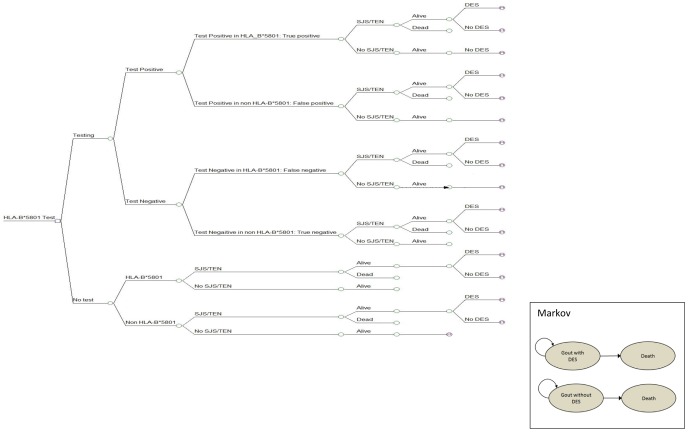
Decision analytic model. **SJS** =  indicated Stevens-Johnson syndrome; **TEN** =  toxic epidermal necrolysis; **M** =  markov model.

A decision analytic model was developed to evaluate the clinical and economic outcomes of testing for HLA-B*5801 genotype before allopurinol administration compared with usual care. The use of Markov model is needed to reflect long-term outcomes because some surviving SJS/TEN patients may suffer from long-term sequelae (**Figure 1**). A cost-utility analysis was conducted in accordance with pharmacoeconomic guideline in Thailand [21]. The results were presented as an incremental cost per quality-adjusted life years (QALYs). Time horizon was life-time and the discount rate of 3% was applied to both cost and outcome [21], [22]. We conducted a cost-utility analysis using a decision tree combined with Markov models using the societal perspective to calculate the expected costs and outcomes in allopurinol patients aged 30 years old and older, which is in the most common age range (30–39 years old) of SJS/TEN cases reported to Thai Health Product Vigilance Center (HPVC) [23]. The hypothetical cohort of 1,000 patients requiring allopurinol for preventing or treating gouty patients with hyperuricemia, and tophaceous gout was entered into the model. In the usual care strategy, all patients received standard care of allopurinol without genetic testing. In contrast, in the genetic testing strategy, patients underwent HLA-B*5801 testing before allopurinol administration. Those who were identified as positive for HLA-B*5801 allele received the alternative drug (probenecid), whereas all other negative testing received allopurinol. According to the American College of Rheumatology (ACR) guideline, febuxostat is recommended as the alternative choice for management of gout [14], however this drug is not available in Thailand. Therefore, we decided to use probenecid as an alternative in our model because of several reasons. First, probenecid has been available in Thailand for a long time and most physicians are familiar with this drug. Second, this drug was recommended for patients with gout where allopurinol was not tolerated, or contraindicated [10], [11], [14], [15], [17]. Third, there were evidences supporting that the efficacy of probenecid is similar to that of allopurinol in patients with normal renal function [10], [11], [17], [24]. Therefore, we assumed that all patients received probenecid as an alternative in this model have normal renal function. We did not include cost of renal function test in the model because renal function test was a routine laboratory test recommended for all gout patients in Thailand [10]. To confirm whether the efficacy of probenecid and allopurinol is comparable, we conducted a systematic review and meta-analysis of randomized controlled trials directly comparing both products and found no evidence demonstrating any statistical and clinical differences in terms of gouty attack rate and serum uric acid level (**Appendix S1**). For gout with mild to moderate renal insufficiency, benzbromarone was recommended as an alternative drug [10]–[12], [15]. Although the efficacy of benzbromarone is similar to allopurinol, it is not commonly used in Thailand and other countries because of the concern of drug-induced hepatotoxicity [10]–[12], [15].

A Markov model was constructed to estimate long-term effects of HLA-B*5801 testing, which consisted of 2 health-states including gout (with and without long-term complications) and death. The model mimicked what would happen in real life as patients were followed till death. Because some of these patients may suffer from long-term sequelae of SJS/TEN, we took into account the probability of having long-term ocular complication. Based on a study conducted in Asian population by Yip et al. [6], 37.6% of SJS/TEN patients developed late ocular complications. The model took into account the sequelae associated with SJS/TEN e.g. corneal scars and visual loss which had been reported as disabling permanent sequelae [25].

### Likelihood of Events

The probabilities of clinical events used in the decision model are shown in **Table 1**. The incidence of allopurinol-induced SJS/TEN is varying from 0.79 to 2.41 per 1000 persons (0.079 to 0.241%) with average of 1.6 per 1000 persons. This incidence was derived from the previously published study conducted by Limkobpaiboon et al [26] as they represented Thai subjects. The prevalence of HLA-B*5801 genotyping was 15% and ranged from 12% to 18% [27], [28]. The prevalence of each study was calculated from allele frequency reported of HLA-B*5801 in Thai population from Allele Frequency Net Database (AFND) [28]. The pooled prevalence was obtained from a meta-analysis of HLA-B*5801 prevalence in the 4 studies of Thai population [27], [28].

**Table 1:** Input parameters.

Parameters	Base case	Range (min-max)	Source(s)
**Epidemiological data**			
Incidence of allopurinol-induced SJS/TEN in Thai population	0.0016	0.00079–0.00241	[26]
Prevalence of HLA-B*5801 in Thai population	0.15	0.12–0.18	[27], [28] a
Probability of death among SJS/TEN	0.1134	0.0503–0.1765	[26]
Probability of DES in SJS/TEN patients	0.3760	0.2882–0.4704	[6]
**Test characteristics**			
Sensitivity	1.000	NA	DMSC
Specificity	1.000	NA	DMSC
**Gene-disease association**			
Association between HLA-B*5801 and allopurinol-induced SJS/TEN in Thai population	348.33	19.15–6336.88	[8], [20]
**Costs (THB, year of costing: 2013)**			
***Direct medical care costs***			
Annual cost of SJS/TEN management	15440.275	339.40–129492.25	[38]
Cost of HLA-B*5801 testing	1,000	800–1,200	DMSC
Annual drug cost of allopurinol	411.99	137.33–823.99	[39]
Annual drug cost of probenecid (alternative)	1,277.50	1,277.50–2,555.00	[39]
Annual cost of gout management	7,512	149–474,254	BCRH b
Annual cost of DES management	4,043.90	3,235.12–4,852.68	Expert
***Direct non-medical care costs***			
Cost of transportation for patient with SJS/TEN	151.93	121.54–182.32	[22], [38]
Cost of transportation for relative of patient with SJS/TEN	1,030.09	824.07–1,236.10	[22], [38]
Cost of additional food for relative of patient with SJS/TEN	379.44	303.55–455.33	[22], [38]
Cost of transportation for patient with gout	1,194.63	1,004.09–1,385.16	[22]
Cost of additional food for relative of patient with gout	440.06	385.03–495.08	[22]
Cost of transportation for patient with DES	607.72	486.18–729.26	[22]
Cost of additional food for relative of patient with DES	223.86	179.09–268.63	[22]
**Outcomes**			
Life expectancy of Thai population (at 30 years old)	42.7	-	[41]
Utility value of patient with gout	0.71	0.638–0.736	[31]
Utility value of patient with gout and dry eye syndrome	0.48	0.40–0.56	[31]–[33] c
**Discount rate** (% per year)	3	0–6	[42]

a Data from meta-analysis.

b Calculated from Buddhachinaraj Regional Hospital (BCRH) database using ICD-10 for identification of patients.

c Calculated from utility of patients with DES and utility of gout patients using multiplicative method.

SJS = Stevens-Johnson syndrome; TEN = toxic epidermal necrolysis; THB = Thai baht; DES = dry eye syndrome; DMSC = Department of Medical Sciences, Thailand.

The probability of allopurinol-induced SJS/TEN in patient with and without HLA-B*5801 was calculated from the following formulae using conditional probabilities [29] where I_a_ =  incidence of allopurinol-induced SJS/TEN; I_1_ =  incidence of allopurinol-induced SJS/TEN in patient with HLA-B*5801; I_0_ =  incidence of allopurinol-induced SJS/TEN in patient without HLA-B*5801; P =  prevalence of HLA-B*5801 in Thai population; and OR =  odds ratio of the association between HLA-B*5801 and allopurinol-induced SJS/TEN:



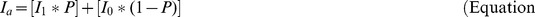
(Equation\;1)




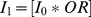
(Equation\;2)However, as we know neither the incidence of allopurinol-induced SJS/TEN in patients with nor without HLA-B*5801 specifically, but we know the incidence of allopurinol-induced SJS/TEN (*I_a_*) [26], prevalence of HLA-B*5801 in Thai population (P) [27], [28], and the association between HLA-B*5801 and allopurinol-induced SJS/TEN (OR) [8], [20]. Thus, according to equation 1 and 2, then the incidence of allopurinol-induced SJS/TEN in patients without HLA-B*5801 can be calculated as below.



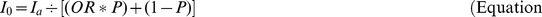
(Equation\;3)Based on a study in 81 Thai patients, the strong association between HLA-B*5801 and allopurinol-induced SJS/TEN was found. The odds ratio (OR) was 348.33 (95% confidence interval (CI) of 19.15–6336.88) [8], [20]. After calculation using the formulae in equation 3 and 2, the incidences of allopurinol-induced SJS/TEN in patients with and without HLA-B*5801 were 0.01049 (10.49 per 1,000) and 0.00003 (0.03 per 1,000), respectively.

The probability of death attributable to allopurinol-induced SJS/TEN was based on a previously published study [26] conducted in a tertiary hospital in Bangkok, Thailand. Of 136 severe cutaneous adverse reactions (SCAR) patients included in this study, 97 patients were diagnosed as SJS/TEN and 11 died (11.34%). We used 11.34% as case-fatality rate for patients with SJS/TEN. The transition probability from no-events to death was based on the age-specific mortality rate (ASMR) for Thai population [30]. In this analysis, we assume that the probability of developing SJS/TEN is zero when the alternative drug (probenecid) is used since there is no evidence of SJS/TEN cases caused by probenecid reported to Thai HPVC [23].

#### Utility

The model took into account the quality of life of patient with gout and sequelae associated with SJS/TEN, e.g. corneal scars and visual loss which had been reported as disabling permanent sequelae [25]. There was no study and utility data in Thai patients with gout and dry eye syndrome. The utility of patients with gout was estimated from a multi-country study conducted by the Institute of Medical Science that assessed the health-related quality of life of patients with gout in Europe [31]. The health-related quality of life was measured from 417 European patients by the EQ-5D. The average EQ-5D value with gout (including both treated and untreated) was 0.710 (95% CI 0.638–0.736)[31].The utility of dry eye syndrome (DES) had been measured using time trade off (TTO) technique in 2 published studies. The utility values were meta-analyzed using a random-effects model [32], [33]. The summary utility value of DES was 0.68 (95%CI 0.57–0.79). Since there was no data for utility of gout patients with DES in any country, we estimated the utility using multiplicative approach as recommended by Ara et al [34].Thus, the utility in gout patients with dry eye syndrome used in this study was 0.48 (95%CI 0.40–0.56) [31]–[33].

#### Test Characteristics

**Figure 2:**
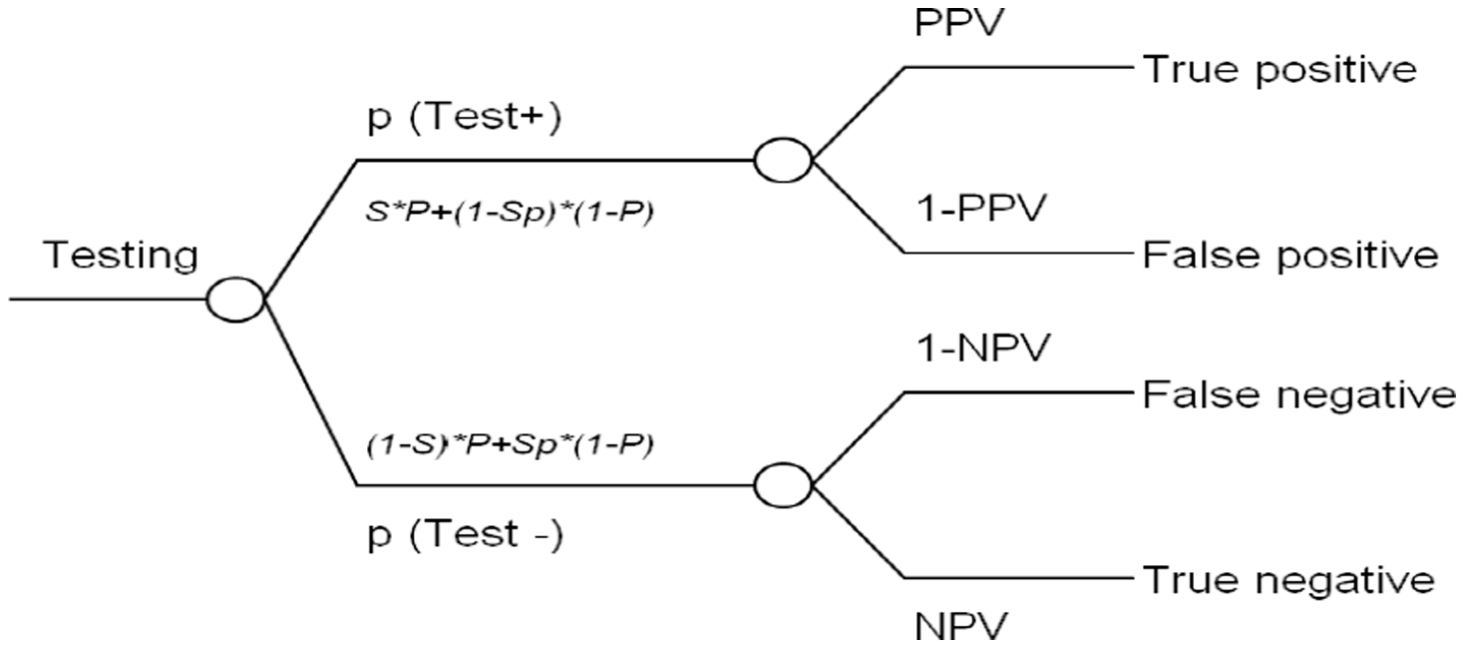
Decision tree for calculating the effectiveness of diagnostic test. **p** = probability; **P** = prevalence; **S** = sensitivity; **Sp** = specificity; **PPV** = positive predictive value; **NPV** = negative predictive value.



The HLA-B*5801 genetic testing, evaluated in this study, was developed and test by Department of Medical Sciences (DMSC), Thailand. Both sensitivity and specificity of this test were 100% [DMSC, personal communication]. To incorporate test characteristics into the decision tree model, we used the conditional probabilities which were calculated using Bayesian analysis [29] as shown in **Figure 2** where *P* =  prevalence, *S* =  sensitivity, and *Sp* =  Specificity. Positive predictive value (PPV) is the proportion of patients who truly have HLA-B*5801 variant allele among those with positive test results; while negative predictive value (NPV) is the proportion of patients who truly have no HLA-B*5801 variant allele given the negative test results. The PPV and NPV can be calculated using the following formulae [35] (equation 4 and 5) and shown in **Figure 2** which was previously illustrated in another model [36].

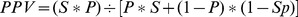
(Equation\;4)




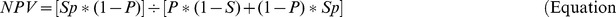
(Equation\;5)


### Costs

The costs included (i) direct medical care costs e.g. costs of SJS/TEN management, costs of gout management, costs of HLA-B*5801 testing, costs of allopurinol drug, and costs of alternative drug (probenecid), and (ii) direct non-medical care costs e.g. cost of transportation and additional food for patient who visiting hospital for gout, dry eye syndrome (DES) and SJS/TEN. We did not incorporate indirect cost because incorporation of productivity gained could result in double counting with utility values included in cost-utility analysis [21].

All costs were estimated for Thai settings from literature providing cost estimates and from a retrospective database analysis. The costs were converted at the rate of 31Thai Baht (THB) per 1 USD [37]. Cost of SJS/TEN management was obtained from the previous study which calculated from 151 patients diagnosed with SJS/TEN (ICD-10 codes: L51.1 and L51.2) using the database of a 1000-bed tertiary care hospital located in the northern part of Thailand [38]. The average cost for SJS/TEN management was 15,440 THB (USD 498). The annual cost of allopurinol drug and alternative drug (probenecid) were retrieved from Drug and Medical Supplies Information Center (DMSIC) database [39] and calculated as 412 THB (USD 13) and 1,278 THB (USD 41), respectively. Median costs of allopurinol (100 mg/tab) and probenecid (500 mg/film coated tab) were 0.376 THB (USD 0.012) per tab and 1.747 THB (USD 0.056) per tab, respectively. Cost of HLA-B*5801 testing was obtained from Department of Medical Sciences (DMSC) with the cost of 1,000 THB (USD 32) including fee charge of OPD visiting. The annual medical care cost of gout management was obtained from 477 patients diagnosed with gout (ICD-10 codes: M10) using the database from year 2008-2010 of a 1000-bed tertiary care hospital located in the northern part of Thailand. Costs of allopurinol and probenecid were excluded from the total cost of gout management, and then the annual medical care cost of gout management was calculated using ratio of cost to charge (0.77) [38]. The average annual medical care cost of gout management was 7,512 THB (USD 242).

Direct non-medical costs included cost of transportation, and additional food cost for patient who visiting hospital for SJS/TEN, gout, and DES (a long-term sequelae of SJS/TEN). These costs were based on the standard cost lists for health technology assessment in Thailand [22] and number of visit for gout and DES. These standard costs were collected by face-to-face interview of 900 patients from all areas around Thailand. All costs were converted to 2013 value using the medical-care consumer price index (CPI) [40].

### Base-case Analyses

The results of the cost-utility analysis were presented as an incremental cost per quality- adjusted life year (QALY) gained for HLA-B*5801 testing versus no testing before allopurinol administration. Quality-adjusted life year (QALY) gained was defined in terms of the gained in years of quality life. This can be calculated by multiplying of expected probabilities of each strategy, life expectancy, and utility. Life expectancy (LE), the average number of years that a person of a given age is expected to remain alive, was obtained from Ministry of Public Health, Thailand [41]. In addition, the number of SJS/TEN cases and death-related SJS/TEN avoidance were also presented.

### Sensitivity Analyses

To determine the robustness of our analysis, we conducted sensitivity analyses including one-way, scenario, and probabilistic sensitivity analyses (PSA).An alternative choice for gout management such as benzbromarone was used in a scenario sensitivity analysis. We also varied the age of cohorts simulated our analyses since SJS/TEN were reported in cases with a wide range of age.

A series of sensitivity analyses were performed to investigate the effects of altering parameters within plausible ranges including epidemiologic data, test characteristics, gene-disease association, and costs (**Table 1**). Discount rate from 0–6 was used in the model [42]. The results of one-way sensitivity were presented using a tornado diagram. The threshold for being cost-effective is defined at 1.2 times Gross National Income (GNI) per capita or 160,000 THB (USD 5,161) [43].

In addition, a probabilistic sensitivity analysis (PSA) was conducted to examine the effect of all parameters uncertainty simultaneously using a second order Monte Carlo simulation performed by Microsoft Excel 2010 (Microsoft Corp., Redmond, WA). All input parameters were assigned a probability distribution to reflect the feasible range of values the each parameter could attain (**Table 1**). The rational for distributional assumption selection for each variable has been given detail elsewhere [44], [45]. The beta distribution was chosen for probability variables, utilities, and test characteristics parameters, which were bounded by zero to one, gamma distribution and log-normal were used for all cost parameters and genetic-outcome association parameter (risk ratio; RR), respectively. In addition, the expected net monetary benefit (NMB) was calculated for each iteration. The results were also presented as cost-effectiveness acceptability curves.

## Results

### Base-case Analysis

In the base-case analysis, genetic testing for HLA-B*5801 before allopurinol administration led to an absolute decrease in the incidence of SJS/TEN of 1.57 cases per 1,000 exposures, and a decrease in the incidence of death attributable to SJS/TEN of 0.18 cases per 1,000 exposures (**Table 2**). Based on a hypothetical cohort of 1,000 patients as given in table 2, the incremental total cost was 923,919 THB(USD 29,804) and incremental QALY was 5.89, thus the ICER was156,937 (USD5,062) per QALY gained. Compared with usual care, the genetic testing strategy before allopurinol administration was a cost-effective intervention, based on a standard cost-effectiveness threshold of 160,000 THB (USD 5,161) per QALY gained in Thailand (year 2013).

**Table 2:** Outcome measures following base-case analyses^a^.

Outcome measure	Genetic testing	Usual care	Difference b
**Incidence** (per 1,000 exposures)			
SJS/TEN	0.0256	1.6000	−1.574 (98.37%) c
Death in SJS/TEN cases	0.0029	0.1814	−0.179 (98.40%) c
**Cost-effectiveness analysis** (n = 1,000)			
Costs (THB, year of costing 2013)			
***Direct medical care costs***	**190,614,332.36**	**189,684,493.13**	**929,839.22**
Cost of HLA-B*5801testing	1,000,000.00	0	1,000,000.00
Annual drug cost	9,859,037.22	9,886,186.08	−27,148.86
Cost for SJS/TEN	395.46	24,704.44	−24,308.98
Annual cost for gout	179,754,073.32	179,721,980.15	32,093.17
Annual cost for DES	826.36	51,622.47	−50,796.12
***Direct non-medical care costs***	**39,116,395.55**	**39,122,315.90**	**−5,920.35**
Cost for SJS/TEN visiting	39.99	2,498.34	−2,458.35
Cost for gout visiting	39,116,185.63	39,109,201.85	6,983.78
Cost for DES visiting	169.93	10,615.56	−10,445.63
**Total costs**	**229,730,727.91**	**228,806,808.89**	**923,919.02**
**Quality-adjusted life year gained (QALY)**	**16,989.49**	**16,983.60**	**5.89**
**Incremental cost per QALY (THB)**	**156,937.04**		

a Base-case analysis is the patient who is 30 year old which analyzed in societal perspective.

b Calculated by values of genetic testing minus usual care.

c Percentage of preventable cases when doing genetic testing before allopurinol administration compared with usual care.

### Sensitivity Analyses

**Figure 3:**
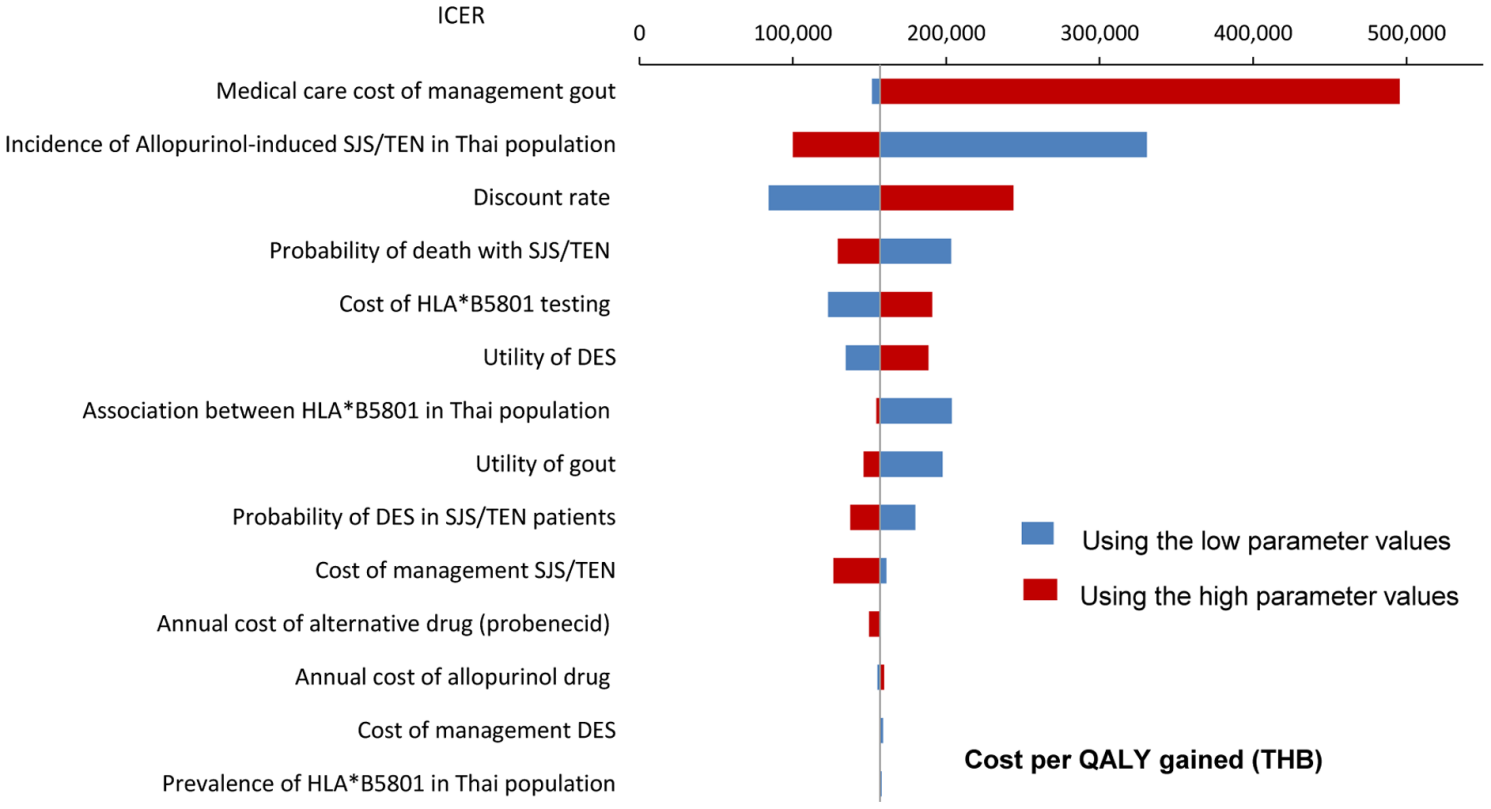
Tornado diagram showing a series of one-way sensitivity analyses comparing genetic testing and usual care. The horizon bars represent the range of the incremental cost-effectiveness ratio (ICER) for one-way sensitivity over the range of parameters. The wider the horizon bar, the more uncertainty that parameter introduces. The vertical line represents the base-case ICER. X-axis indicated the ICER. THB = Thai baht. The low and high parameter values are based on the range specified in Table 1.

A series of one-way sensitivity analyses of other parameters were shown in **figure 3**. The uncertainty in medical care cost of gout management has the largest influence on the ICER. When cost of gout management was varied from 149 to 474,254 THB (USD 4.8 to 15,298), the ICER was 151,594 to 495,645 THB (USD 4,890to 15,989) per QALY gained, respectively. Besides, incidences of allopurinol-induced SJS/TEN, discount rate, and probability of death with SJS/TEN in Thai population also have the large influence on the ICER. When the benzbromarone (median cost/tab: 6.95 THB or USD 0.224) was used as alternative drug instead of probenecid (median cost/tab: 1.75 THB or USD 0.056), ICER was shifted from 156,937 to 156,989 THB (USD 5,062to 5,064) per QALY gained. When the age at start was varied from 25 to 50 years old, the ICER was changed from 148,708 to 221,803 THB (USD 4,797 to 7,155) per QALY gained.

**Figure 4:**
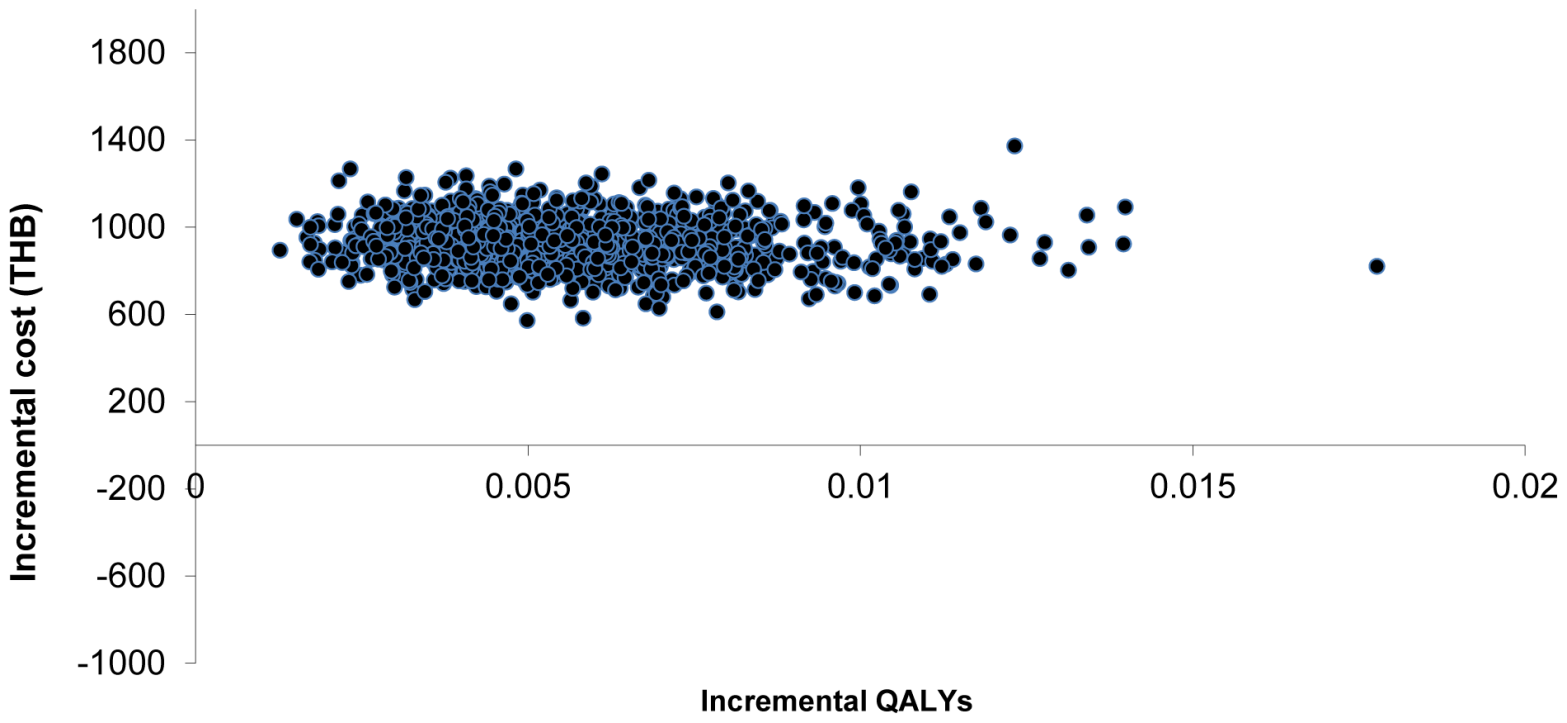
Cost-effectiveness scatter plot. Each point represents incremental cost (year 2013 values) and Quality adjusted life year gained (QALYs) between genetic testing and usual care from Monte Carlo simulation with varying model parameters.

**Figure 5:**
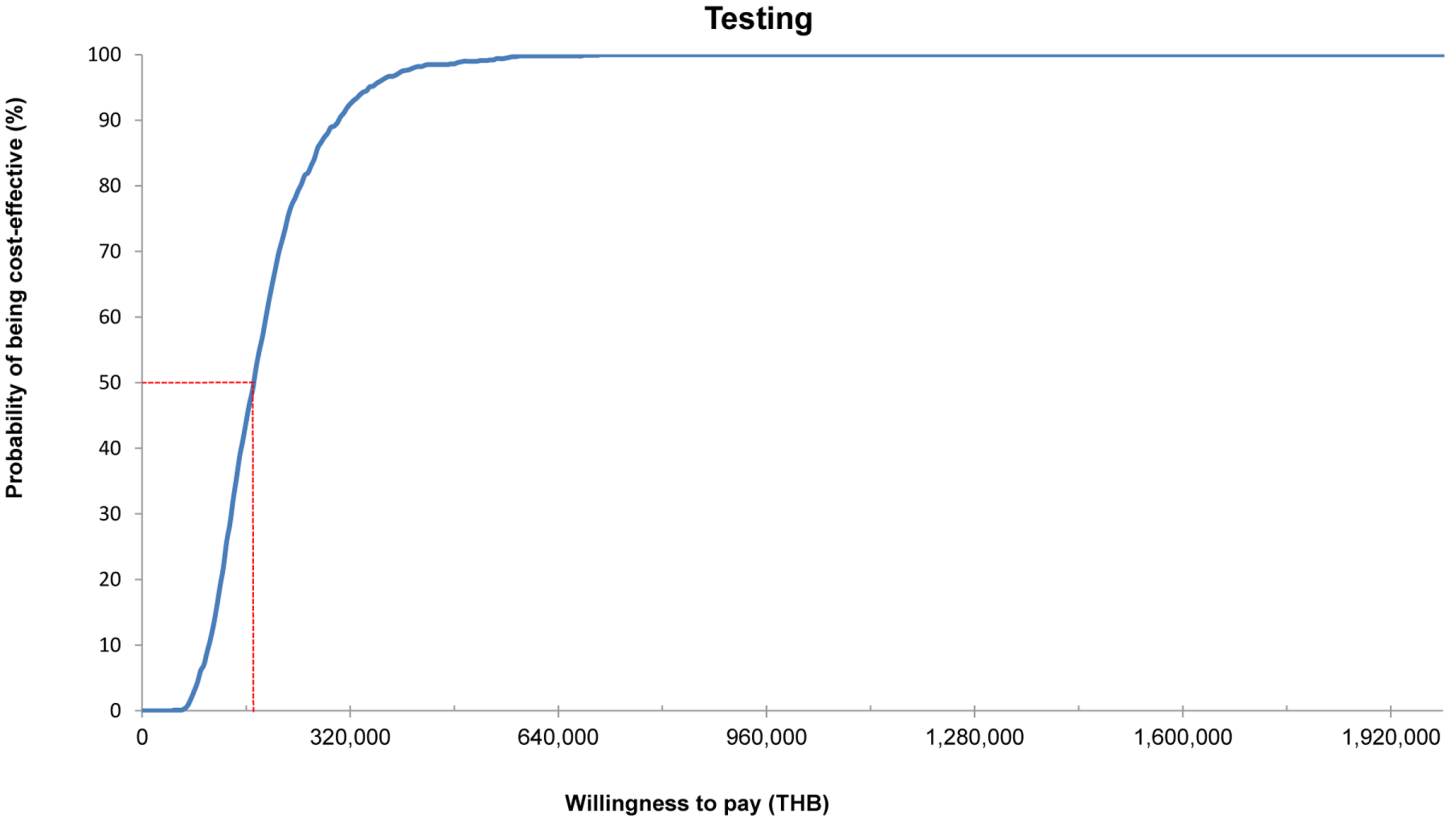
Cost-effectiveness acceptability curve. The curves provide the probability of the different strategy being the most cost-effective option at any willingness to pay value for an additional quality -life year gained for genetic testing.

In the probabilistic sensitivity analysis, genetic testing increased both costs and QALY in all iterations (**Figure 4**). At the threshold of 160,000 THB (USD 5,161) per QALY gained, 49.4% of the iterations were cost-effective (**Figure 5**).

## Discussion

Our analysis suggests that performing HLA-B*5801 testing prior to allopurinol administration reduces the number of SJS/TEN cases and may prevent death attributable to SJS/TEN in patients with gout. To the best of our knowledge, this is the first economic evaluation study evaluating pharmacogenetic testing of HLA-B*5801. The information derived from our study will be used as part of crucial information for policy decision making.

Even though there is no direct value of the incidence of allopurinol-induced SJS/TEN in patients with HLA-B*5801 allele in Thailand, we believe that the calculated incidence is likely to be highly valid. The incidence of allopurinol-induced SJS/TEN in Thailand was estimated as 0.16% [26]. The overall odds ratio of the association of the HLA-B*5801 gene and allopurinol-induced SJS/TEN in Thai was based on a meta-analysis study as 348 [8].The calculated incidence of allopurinol-induced SJS/TEN among subjects with HLA-B*5801 gene was based on highly internal valid sources, leading to the reliable estimate.

We believe that our findings are contextually relevant to the Thai health care system for several reasons. Firstly, we used local data in the analysis, making the results highly applicable to local context. All cost data were acquired from reliable sources i.e. database of patients with SJS/TEN in Thailand and Drugs and Medical Supplies Information Center (DMSIC), Ministry of Public Health. Moreover, our study was conducted in accordance with the Pharmacoeconomic guideline in Thailand [21], [46]. The societal perspective undertaken in our analysis was the most widely recommended perspective.

A number of assumptions used in our study deserved further discussion. First, we assumed that there is no difference in terms of both therapeutic benefits and quality of life between patients receiving allopurinol or probenecid. This might not be necessarily true in all circumstances. Second, we assumed that all patients receive probenecid as alternative, implying that all patients have gout without renal complication. In fact, an alternative drug for gout with mild to moderate renal complication is benzbromarone. However, this drug is rarely used in Thailand and may produce hepatotoxicity. Although febuxostat was recommended as the first alternative choice for patients who hypersensitivity to allopurinol in various countries [11], [12], [14], [15], we did not choose this drug in the model because it was not available in Thailand.

Our study reveals that the cost-effectiveness findings are sensitive to a number of factors, implying a need to consider them seriously while evaluating cost-effective analysis. Those factors include medical care cost of gout management, incidence of allopurinol-induced SJS/TEN, probability of death with SJS/TEN in Thai population, and cost of genetic testing. It is very important to consider the impreciseness of these parameters as key variable explaining the findings. For the use of cost-effectiveness analysis in policy decision making, policy need to take this into consideration to make informed decision.

We believe that our study finding is highly relevant to those countries with high prevalence of HLA-B*5801. The prevalence of such gene was reported as high as 5.5–15% in Thai and Han Chinese populations, while the prevalence was as low as 0.6% and 0.8% in Japanese and European population [28]. The implication is that a genotypic testing of the HLA-B allele may be potentially cost-effective from the societal viewpoint in only countries with large number of subjects at risk such as those from Southeast Asian countries and Han Chinese countries or those with high prevalence of HLA-B*5801.

It is important to note that there are other issues relevant for policy decision making that have not been considered in our analysis yet. First, it is possible that the cost of genetic testing might be lowered if the larger numbers of test kits are purchased. Genetic testing will be more cost-effective if such cost is lowered. Second, currently, there is a test kit for genetic testing as all-in-one, which included HLA-B*1502, HLA-B*5801, and others. The value for money of such all-in-one testing has not been evaluated in this study. However, it is highly potential that the cost-effectiveness value of the test will be better given the broad range of benefits. Lastly, it must be noted that our analysis has not determined affordability issue and feasibility for implementing such genetic testing. In order to implement such test, there is strong need for ensuring clinicians to have full understanding of genetic test and its implication.

## Conclusion

In summary, the genetic testing for HLA-B*5801 before allopurinol administration is considered a highly potential cost-effective intervention in Thailand. The findings are sensitive to a number of factors. In addition to cost-effectiveness findings, consideration of other factors including ethical, legal, and social implications is needed for an informed policy decision making.

## Supporting Information

Appendix S1 **Comparison of efficacy of allopurinol and probenecid: a systematic review of randomized controlled trials.**
(PDF)

Checklist S1 **CHEERS Checklist.**
(PDF)
