# Supplementary material for: Cost-Effectiveness Analysis of HLA-B*5801 Testing in Preventing Allopurinol-Induced SJS/TEN in Thai Population
Source: PLoS One. 2014 Apr 14;9(4):e94294. doi: 10.1371/journal.pone.0094294 (PMC3986079; doi:10.1371/journal.pone.0094294)
Supplement: Appendix S1 — Comparison of efficacy of allopurinol and probenecid: a systematic review of randomized controlled trials. (PDF) [file pone.0094294.s001.pdf]

APPENDIX S1

Comparison of efficacy of allopurinol and probenecid: a systematic review of randomized controlled trials

METHOD

Data sources and Searching strategy

The following databases were systematically searched: MEDLINE, EMBASE, Cochrane CENTRAL. Databases were searched from their inception to March 4, 2014. For the search strategy, we used the Medical Subject Headings (MeSH) 'allopurinol', 'probenecid', and 'gout' with slight modifications based on the sources (search strategy was shown in Appendix 1). References of initially identified articles were examined to identify additional studies that met the selection criteria.

Study selection

The inclusion criteria were studies that: (i) compared allopurinol with probenecid , (ii) reported the number (or percentage with total number of patients) of outcomes (iii) studied in human.
There was no language and study design restriction. Studies that were not original articles such as comments, letters, reviews, meta-analyses, guideline, case reports, surveys or editorials were excluded. Studies from the same population (duplicate studies), studies not reporting effect-estimates or with insufficient information to compute effect estimates were also excluded.

RESULTS

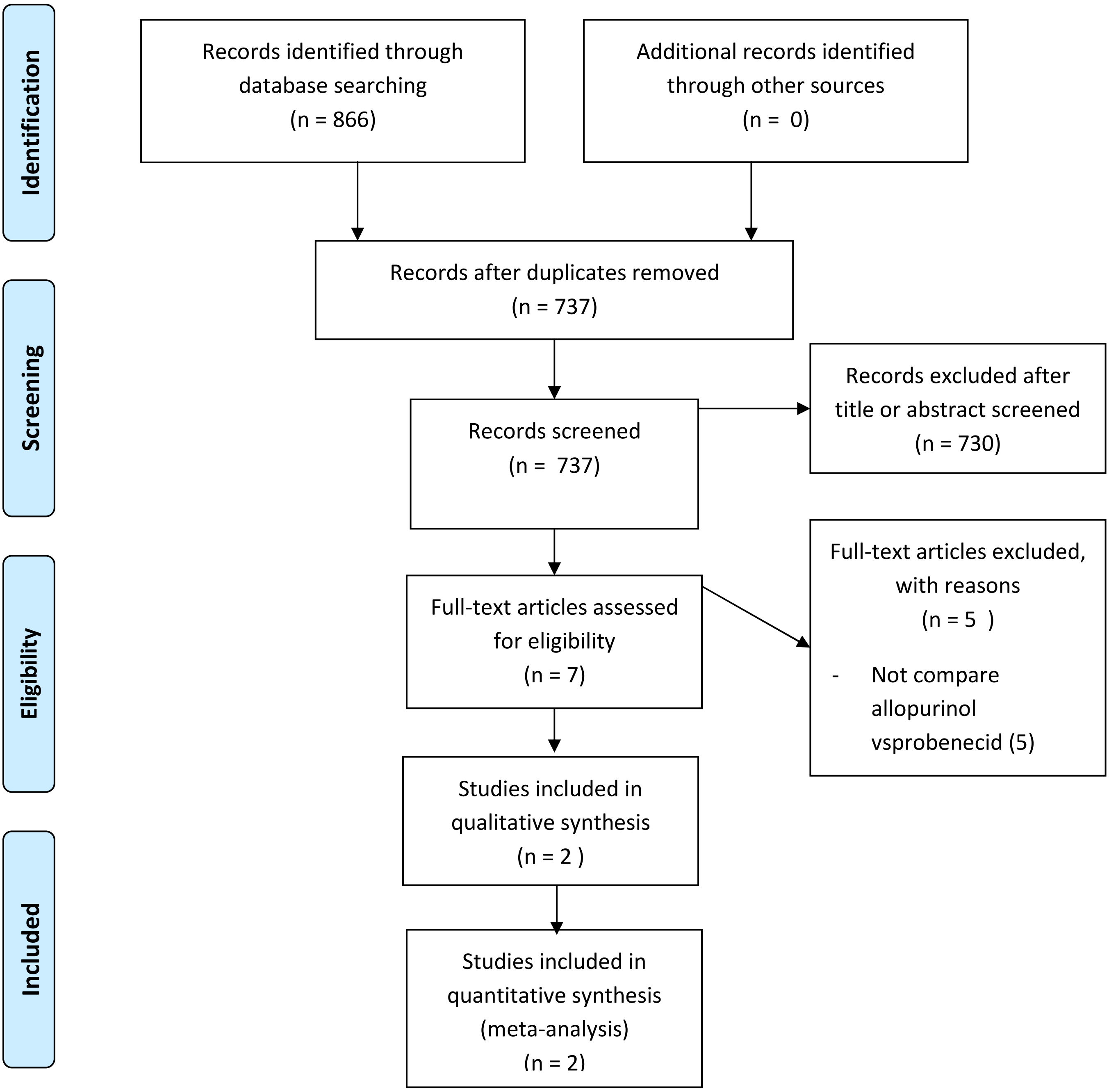

Figure 1: Flow Diagram of study selection

Excerpts of relevant sections from the included articles

Scott 1966

TABLE II
EFFECT OF TREATMENT ON SERUM URIC ACID LEVEL (mg./100 ml.)

Group	I Allopurinol			II Uricosuric Treatment		
	Before Treatment	After 2 Weeks	At Last Estimation	Before Treatment	After 2 Weeks	At Last Estimation
Mean	9·3	5·8	4·7	8·5	6·3	5·2
Range	7·5-10·6	4·5-6·9	2·6-5·5	7·5-11·7	3·9-10·8	3·8-7·3

commenced. In those who became free of symptoms, colchicine was withdrawn several months after the last attack of gout, but it is anticipated that treatment with either allopurinol or uricosuric drugs will be continued indefinitely in the absence of any toxic effects. No special diet was prescribed for these patients. In accordance with our usual practice they were told which foods contain a high purine content, and they were advised to avoid an excessive intake of these foods and of alcohol.

Duration of Follow-up

The mean duration of follow-up for patients taking allopurinol has been 18·6 months (range 10 to 23), and for those taking probenecid 19·6 months (range 11 to 24). After initial assessment, patients have been seen at intervals of 2 weeks, 1 months, 2 months, and 3 months; and at 3-monthly intervals thereafter.

Results

(1) Frequency of Acute Gout.—It is difficult to be precise about the frequency or severity of attacks of gout. All patients were having recurrent attacks before treatment was started (Table I) and the overall effect has been to reduce the number of attacks in both treatment groups. As mentioned above, all patients were given colchicine. Of the twenty patients receiving allopurinol, nine have had no further gout since starting treatment, six have had one further attack between 1 and 9 months after starting treatment, four had two further attacks 2 and 15 months after starting treatment (one mostly in the early months), and one patient continued to have severe attacks for 6 months after starting treatment but has been free of gout since. Of the seventeen patients taking uricosuric treatment, eight have had no further gout after starting treatment, six had one further attack occurring between 2 weeks and 15 months after starting treatment (one of these had temporarily stopped his tablets because of pain from his duodenal ulcer), two had two attacks at between 2 weeks and 17 months after starting treatment, and one had three further attacks occurring between 3 and 5 months after starting treatment.

In both treatment groups, therefore, about half the patients had no further gout after starting treatment and in the others attacks have become much less frequent. Such attacks as did occur in these early months, however, appeared to be of considerable severity.

(2) Tophi.—As will be evident from the description of the patients, most of the subjects in this comparative trial did not have advanced tophaceous gout. In two of the three patients with very small tophi taking allopurinol these have disappeared, and the same has occurred in one of the two patients with tophi taking probenecid.

(3) Serum Uric Acid.—Drug dosage was regulated to some extent to produce a satisfactory fall in the SUA level without trying to go beyond this. Table II shows the mean SUA level in each treatment group (*a*) before treatment was started, (*b*) 2 weeks

Stocker 2008

Table II. Pharmacodynamic effects of oxypurinol and probenecid following administration of allopurinol and/or probenecid for 7 days in healthy subjects (n = 11)[a]

Parameters	Baseline[b]	Allopurinol	Probenecid	Allopurinol and probenecid
C_{UA} (mmol/L)	0.30 ± 0.05	0.16 ± 0.05**	0.13 ± 0.02**,†	0.09 ± 0.02**,††,‡‡
$U_{ex(UA)}$ (mmol/day)	3.7 ± 1.6	1.9 ± 0.8**	4.7 ± 1.5*,††	3.2 ± 1.1††,‡
$U_{ex(Cr)}$ (mmol/day)	14 ± 6	14 ± 3	14 ± 4	13 ± 3
UA/Cr (mol/mol)	0.27 ± 0.05	0.13 ± 0.03**	0.35 ± 0.04*,††	0.23 ± 0.04††,‡‡
$CL_{R(UA)}$ (mL/min)	8.9 ± 4.0	8.0 ± 2.4	26 ± 10**,††	26 ± 7**,††

a Values are expressed as mean ± SD.

b Drug-free conditions.

$CL_{R(UA)}$ = renal clearance of urate; **C_{UA}** = plasma concentration of urate; **UA/Cr** = ratio of urate output to creatinine output; **$U_{ex(Cr)}$** = urinary excretion rate of creatinine; **$U_{ex(UA)}$** = urinary excretion rate of urate; * $p < 0.05$, ** $p < 0.001$ vs baseline; † $p < 0.05$, †† $p < 0.001$ vs allopurinol alone; ‡ $p < 0.02$, ‡‡ $p < 0.001$ vs probenacid alone.

1 mg/ 100 ml = 54.48 mcmol/L = 0.05448 mmol/L
(http://www.endmemo.com/medical/unitconvert/Uric_acid.php)
So, multiplying mmol/L unit by 18.35536 to convert mmol/L to mg/100 ml.

Table 1: Description of characteristics of studies

Characteristics	Scott 1966	Stocker 2008
Study design	Randomized controlled trial	Randomized controlled trial
Study group	Newly diagnosed subjects with gout	Health subjects
Number	20	17
Sex	All were men	8 female, 3 male
Age, mean (range)	Allopurinol 54 years (27 – 78)<br>Probenecid 54 year (38 - 76)	23.5 years (range 21 – 36)
Drug regimen	Allopurinol 300 mg daily but increase where necessary *vs*<br>Probenecid 1 mg daily rising to 2 mg daily after 2 weeks	Allopurinol 150 mg twice daily *vs*<br>Probenecid 500 mg twice daily
Follow up	Allopurinol 10-23 months<br>Probenecid 10-24 months	1 week
Outcome measurement time	2 weeks	1 week
Baseline serum uric acid (mg/dL), mean (SD)	Allopurinol 9.3 (1.55)<br>Probenecid 8.5 (2.1)	Allopurinol 5.51 (0.92)<br>Probenecid 5.51 (0.92)

Outcomes

In this systematic review, two outcomes are used for analysis. The first is gout attack, while the second one is the serum concentration of uric acid.

Gouty attack

Scott 1966 was the only study investigating this outcome. Eleven out of 20 subjects receiving allopurinol had gouty attacks, while 9 out of 17 subjects receiving probenecid had. This translated to risk ratio of 1.03 (95% CI; 0.50, 2.09). It is interpreted that the proportion of patients having gouty attacks is slightly higher in the group of allopurinol compared to the probenecid group but it is not statistically significant.

Below is the analysis

```
csi 11 9 20 17

                 |   Exposed   Unexposed  |      Total
-----------------+------------------------+------------
           Cases |        11           9  |         20
        Noncases |        20          17  |         37
-----------------+------------------------+------------
           Total |        31          26  |         57
                 |                        |
            Risk |  .3548387    .3461538  |   .3508772
                 |                        |
                 |      Point estimate    |    [95% Conf. Interval]
                 |------------------------+------------------------
 Risk difference |         .0086849       |  -.2399282    .2572979
      Risk ratio |          1.02509       |   .5038779    2.085443
 Attr.frac. ex.  |         .0244755       |  -.9846077    .5204856
 Attr.frac. pop  |         .0134615       |
                 +-------------------------------------------------
               chi2(1) =     0.00  Pr>chi2 = 0.9454
```

Serum concentration of uric acid

The serum concentration of uric acid in patients who received either allopurinol or probenecid we summarized in the table below.

Table 2: Effect of allopurinol and probenecid on serum uric acid (mg/dL)

Study	Allopurinol			Probenecid		
	**N**	**Before** Mean (SD)	**After** Mean (SD)	**N**	**Before** Mean (SD)	**After** Mean (SD)
Scott 1966	20	9.3 (1.55)	5.8 (1.2)	17	8.5 (2.1)	6.3 (3.5)
Stocker 2008	11	5.51 (0.92)	2.94 (0.92)	11	5.51 (0.92)	2.39 (0.37)

Then, we pooled the serum concentration of uric acid using DerSimonian and Laird random-effects models. The forest plot of pooled effect size was showed below. The pooled results of the 2 included revealed thatprobenecid may reduce the serum concentration of uric acid by 0.36 mg/dL(95%CI, -.43, 1.15) compared with allopurinol, but notstatistically significant (p =0.373) with low level ofheterogeneity (I^2 = 20%; p = 0.264).

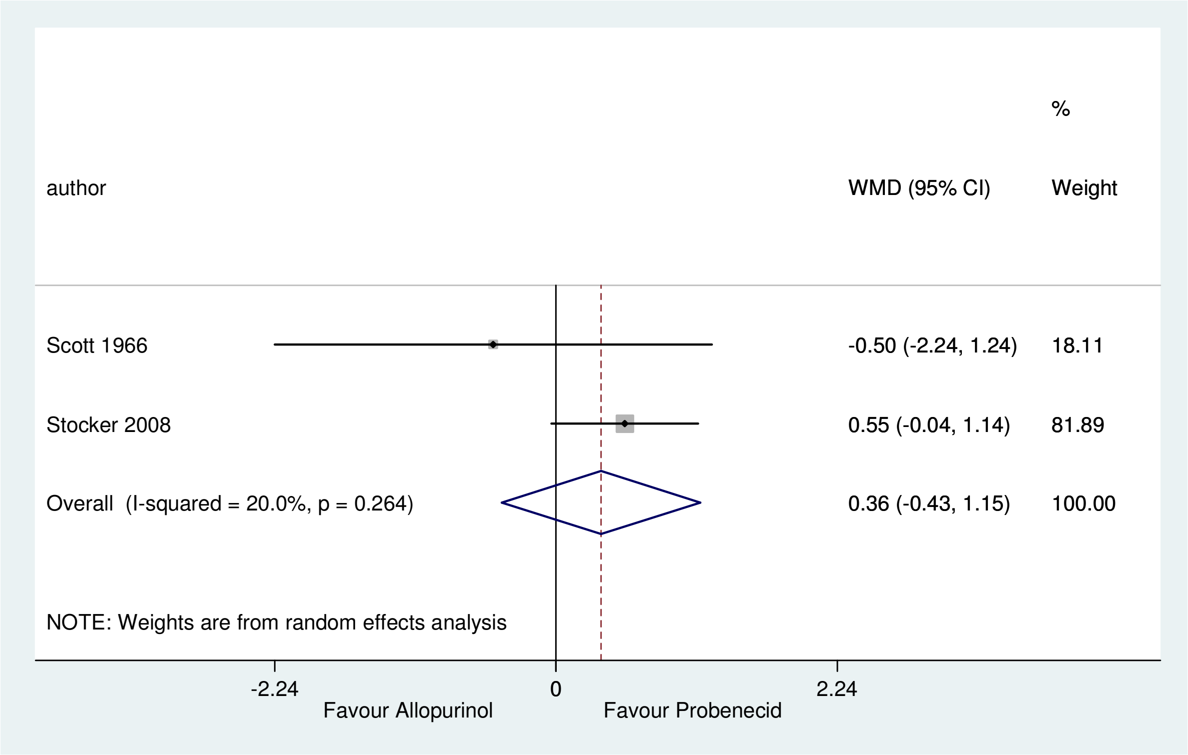

Figure 2: Forest plot of randomized controlled trials assessing the effect of probenecid compared to allopurinol on serum uric acid. The diamond indicates the weighted mean different of serum uric acid (mg/dL) and 95% confidence internal (CI).

CONCLUSION

Current evidence demonstrates that there remains no clear evidence showing the significant differences of clinical outcomes between allopurinol and probenecid.

SEARCHING RESULTS

Database	Strategy			N
Medline	Search	Query	Items found	168
	#12	Search #11 AND #8 AND #9	168	
	#11	Search #1 OR #2 OR #3 OR #4 OR #5 OR #6 OR #7	4561	
	#10	Search #1 OR #2 OR #3 OR #4 OR #5 OR #6 OR #	4559	
	#9	Search gout	13248	
	#8	Search Allopurinol	8457	
	#7	Search Renamid	2	
	#6	Search Benacid	0	
	#5	Search Proben	6	
	#4	Search Benecid	4554	
	#3	Search Bencid	0	
	#2	Search Pondnacid	0	
	#1	Search Probenecid	4554	
EMBASE	'probenecid'/exp AND 'allopurinol'/exp AND 'gout'/exp			688
Cochrane	'probenecid' AND 'allopurinol' AND 'gout'			10
**Total**				**866**
